# Variation in Susceptibility to *Wheat dwarf virus* among Wild and Domesticated Wheat

**DOI:** 10.1371/journal.pone.0121580

**Published:** 2015-04-02

**Authors:** Jim Nygren, Nadeem Shad, Anders Kvarnheden, Anna Westerbergh

**Affiliations:** Department of Plant Biology, Uppsala BioCenter, Linnean Centre for Plant Biology in Uppsala, Swedish University of Agricultural Sciences, Uppsala, Sweden; Washington University, UNITED STATES

## Abstract

We investigated the variation in plant response in host-pathogen interactions between wild (*Aegilops* spp., *Triticum* spp.) and domesticated wheat (*Triticum* spp.) and *Wheat dwarf virus* (WDV). The distribution of WDV and its wild host species overlaps in Western Asia in the Fertile Crescent, suggesting a coevolutionary relationship. Bread wheat originates from a natural hybridization between wild emmer wheat (carrying the A and B genomes) and the wild D genome donor *Aegilops tauschii*, followed by polyploidization and domestication. We studied whether the strong selection during these evolutionary processes, leading to genetic bottlenecks, may have resulted in a loss of resistance in domesticated wheat. In addition, we investigated whether putative fluctuations in intensity of selection imposed on the host-pathogen interactions have resulted in a variation in susceptibility to WDV. To test our hypotheses we evaluated eighteen wild and domesticated wheat taxa, directly or indirectly involved in wheat evolution, for traits associated with WDV disease such as leaf chlorosis, different growth traits and WDV content. The plants were exposed to viruliferous leafhoppers (*Psammotettix alienus*) in a greenhouse trial and evaluated at two time points. We found three different plant response patterns: *i*) continuous reduction in growth over time, *ii*) weak response at an early stage of plant development but a much stronger response at a later stage, and *iii*) remission of symptoms over time. Variation in susceptibility may be explained by differences in the intensity of natural selection, shaping the coevolutionary interaction between WDV and the wild relatives. However, genetic bottlenecks during wheat evolution have not had a strong impact on WDV resistance. Further, this study indicates that the variation in susceptibility may be associated with the genome type and that the ancestor *Ae*. *tauschii* may be useful as genetic resource for the improvement of WDV resistance in wheat.

## Introduction

Wild plant populations are constantly influenced by abiotic and biotic factors. The biotic stresses caused by pathogenic infestations exert a selective pressure on the evolution of defense mechanisms in host plants. The selective forces act mutually and the plant defense triggers a response in the colonizing pathogens. An arms race between the development of virulence in the pathogen and resistance in the host plant evolves. The strong selection imposed upon the other partner in the pathogen-host plant interaction will shape the genetic diversity and evolution of both organisms. The arms race may reach a stable, balanced polymorphism in the host plant-pathogen interaction if there is a negative frequency-dependent selection on the plant, the pathogen or both [1]. This means that when a phenotype such as resistance or virulence is rare in the population, the phenotype is relatively favored by natural selection but when it becomes more common, the fitness decreases and the interaction has reached an equilibrium.

The domesticated crops are often highly susceptible to many pathogens [2–4], whereas the wild crop relatives may be resistant to the same pathogens. The susceptibility in crops may be a consequence of a lack of selection for disease resistance during domestication. Other traits such as increased seed size, increased apical dominance, suppression of natural seed dispersal, loss of seed dormancy and synchronized growth seem to have been favored by the ancient farmers in seed crops and selected from the standing genetic variation in wild crop ancestors [5–7]. Due to early agricultural practices where farmers selectively collected seeds from plants with desirable traits and planted them in their fields, the phenotype of the plants changed over time and much of the genetic diversity was unconsciously left behind in the wild populations. Thus, the genetic bottleneck caused by domestication may have hampered the arms race between the pathogen and the crop, and instead increased the susceptibility in the host. Disease resistance is also widespread in natural plant populations [8] and references therein]. However, diversity of susceptibility and resistance occur both within and among populations as a result of trade-offs (cost of resistance and virulence) and/or spatial variation in intensity of selection in coevolutionary plant-pathogen interactions [9]. By evaluating a number of plant-pathogen interaction studies, Laine et al. [8] found that the diversity in resistance provided a higher protection against pathogens at the population level.

For a better understanding of the response to pathogen infections in crops and their wild relatives as well as the effects of crop domestication on the pathogen resistance we are studying the response in wild and domesticated wheat to *Wheat dwarf virus* (WDV, family *Geminiviridae*, genus *Mastrevirus*).

Bread wheat (*Triticum aestivum* ssp. *aestivum*) is susceptible to WDV and no highly resistant cultivar is known. However, variation in susceptibility has been found among cultivars [10–13], and recently two Hungarian winter wheat cultivars were found to display partial resistance to WDV [14]. WDV disease outbreaks may occur periodically and cause yield losses in most of Europe and in parts of Africa and Asia [14,15]. Recently, WDV has also been detected in Syria [16] and in Iran [17]. The incidence of WDV disease in Swedish bread wheat fields can be up to 90% in severe cases [12]. Like many other geminiviruses WDV causes severe symptoms on host plants including dwarfing, tufting, streaks of leaf chlorosis, and reduced number of spikes that are often sterile and stunted [18]. The virus is transmitted by the leafhopper vector *Psammotettix alienus* (family Cicadellidae). However, virus-free *P*. *alienus* feeding on wheat plants does not cause any visible symptoms [19]. The primary spread of WDV to winter wheat takes place in autumn, when the adult leafhoppers migrate into newly sown fields. *P*. *alienus* completes two to three generations per year. In central Sweden the first adult generation appears in June-July and the second one in August-September [12]. The leafhoppers overwinter as eggs and the first generation of nymphs appears in May. WDV is not transmitted to the eggs and the nymphs of the first instar [20]. WDV may be considered as a grass generalist pathogen since its host range encompasses not only wheat but also several wild grasses and other cereals such as barley, oat and rye [18]. For infection of wheat, wild grasses are of less importance as primary sources compared to cultivated wheat. However, the grasses growing in vicinity to cultivated cereal fields may act as reservoirs of WDV [21].

WDV has a genome of single-stranded circular DNA [22,23]. Five strains of WDV have been described, WDV-A to WDV-E [23]. Wheat-infecting isolates of WDV are usually unable to infect barley and the other way around [24–26]. Most of the WDV isolates from wheat belong to the strain WDV-E, which has a very wide geographic distribution throughout Europe and Asia and with isolates sharing a high genome sequence identity [21,23,25]. However, based on wheat-infecting isolates of WDV a high genetic diversity has been found to be concentrated in some regions of the WDV genome including introns, short and long intergenic regions and the coding region of the replication-association protein Rep A [27]. Interestingly, it has been shown in maize streak virus (*Mastrevirus*) that single nucleotide mutations can lead to major changes in severity of symptoms and host range [28,29]. Presence of genetic variation both within the virus and the host plants is fundamental for their coevolutionary relationship. Moreover, evidence has been found for a correlation between cereal host divergence times (*Triticum*, *Hordeum* and *Aveneae*) and WDV divergence times, indicating coevolutionary arms race between the virus and the host plants [27].

WDV is transmitted to the wheat plants by the leafhopper in a persistent manner [19,30]. The virus is mixed with the saliva of the nymph or the adult leafhopper and is inserted to the phloem fluid when the leafhopper is penetrating the plant tissues with its stylet for feeding [31]. In non-immune plants, viruses move with the phloem stream first to the young leaves and root tips, and then to the older parts, until the plant is systematically infected [32]. The response of the plant is not only dependent on its genotype but also on the age of the plant when it is infected. As the wheat plants grow they gradually acquire resistance to the WDV and when the plant has developed its first node on the stem it has acquired mature resistance [33].

**Fig 1:**
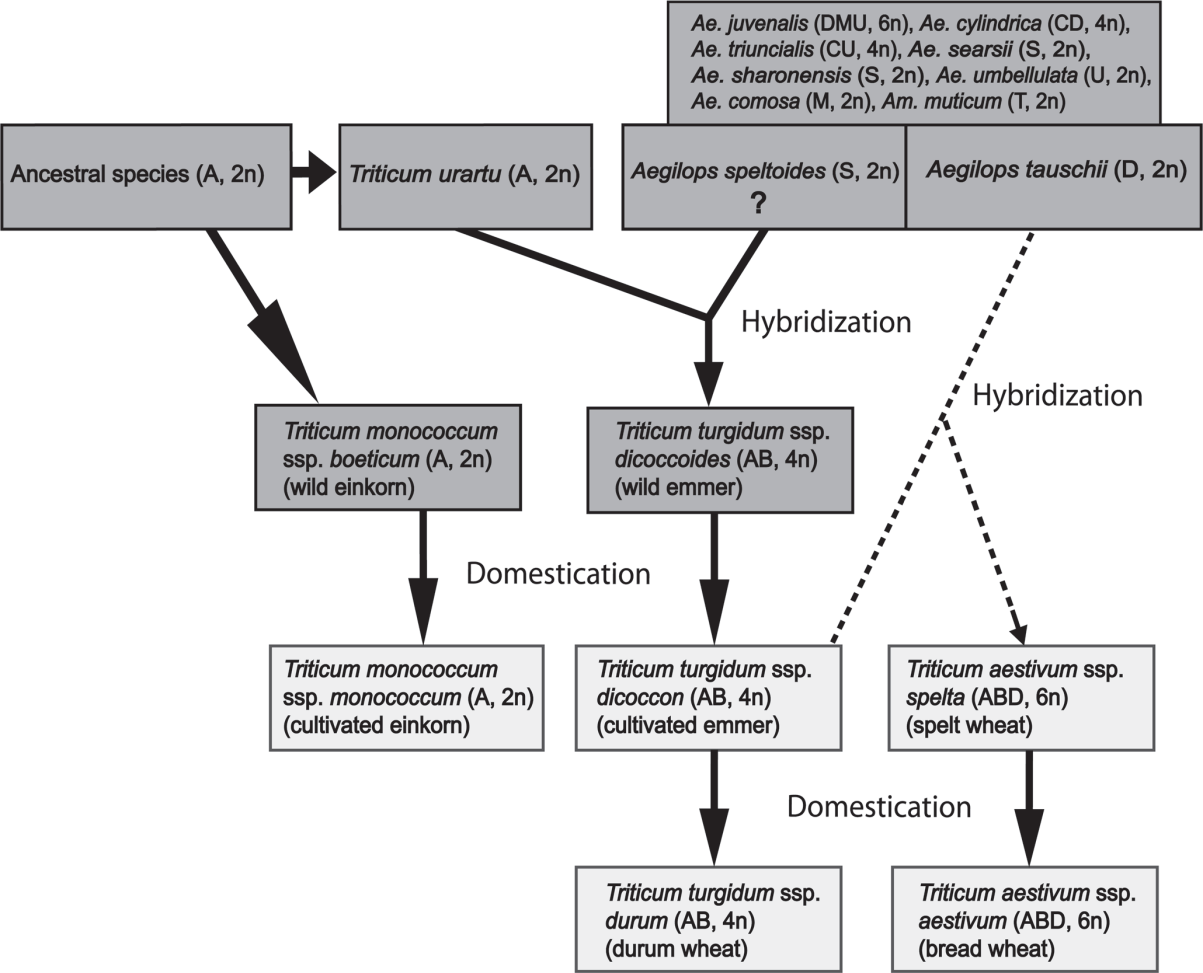
Studied species and their role in the evolutionary history of bread wheat. The figure is based on information described in Zohary et al. [36], Kilian et al. [44], Chantret et al. [45] and Peng et al. [46]. Genome type and ploidy level are given in brackets, LIGHT GREY = wild species, GREY = domesticated wheat. Dashed lines show the most accepted model of the origin of hexaploid wheat where the ancestral species are tetraploid emmer wheat (*Triticum turgidum* ssp. *dicoccon*) and *Aegilops tauschii*. A more recent model is described in Dvorak et al. [43].

Wheat is one of many crops with origin in the Fertile Crescent in Western Asia (Asian part of Turkey, Syria, Lebanon, Israel, Jordan, Iraq and western Iran [34–36]. The first two domesticated forms of wheat were diploid einkorn *T*. *monococcum* ssp. *monococcum*, derived from its wild form *T*. *monococcum* ssp. *boeticum*, and the cultivated tetraploid emmer wheat, *T*. *turgidum* ssp. *dicoccon* derived from wild emmer wheat *T*. *turgidum* ssp. *dicoccoides* (Fig 1). South Eastern Turkey is supposed to be the domestication site of einkorn and emmer wheat [34,37]. The wild emmer underwent previous to its domestication hybridization and polyploidization between two diploids: the A genome donor *Triticum urartu* [38,39] and the suggested B genome donor *Aegilops speltoides* [40,41] (Fig 1). The tetraploid cultivated emmer wheat migrated eastward and hybridized with the wild diploid *Ae*. *tauschii*, the D genome donor [42] and formed the first hexaploid wheat carrying the A, B and D genomes [36]. Whether the first hexaploid wheat was a hulled or free-threshing form has been discussed and additional models for the evolution of hexaploid wheat are presented. In a new model, Dvorák et al. [43] proposed that the ancestral hexaploid wheat was hulled and resulted from a cross between a free-threshing tetraploid wheat, not hulled cultivated emmer wheat, and the *strangulata* subspecies of *Ae*. *tauschii*. Through a mutation in the *Tg* locus, which controls glume tenacity, the ancestral hexaploid wheat evolved into a free-threshing form. In addition to this evolutionary model, spelt is derived from a hybridization between a free-threshing hexaploid wheat and hulled tetraploid emmer wheat.

Besides the domestication process other bottlenecks created by the natural hybridization and polyploidization events in wheat evolution have reduced the genetic diversity of the ancestral genomes in cultivated wheat [47,48].

**Fig 2:**
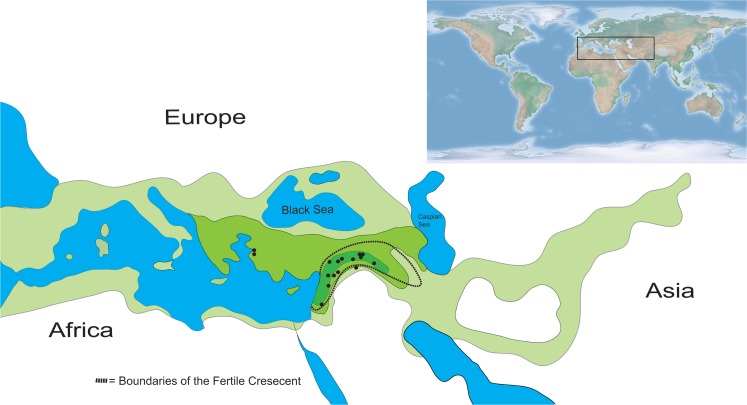
Collection sites of wild and domesticated wheat. ● = *Aegilops*, *Amblyopyrum* and *Triticum* accessions, except spelt and the winter wheat cultivar Tarso, based on SINGER data base, 2009, species richness of *Aegilops* and *Triticum* based on information in Zohary et al. [36] and Van Slageren [51]; LIGHT GREEN = 1–5 species, GREEN = 6–10 species, DARK GREEN = >10 species.

Like all domesticated wheat taxa, their wild relatives, *Aegilops* spp. (22 species), *Triticum* spp. (4 species) and *Amblyopyrum muticum*, are annuals. The wild relatives carry different genomes defined as A, B, C, D, G, M, N, S, T, and U types, and have three different ploidy levels (diploid, tetraploid and hexaploid; [44,49]). They are distributed along the Euro-Asian axis from the Mediterranean region into China and the largest species diversity of wild wheat relatives is found in Western Asia in the Fertile Crescent region [44,50,51] (Fig 2). The wild relatives are adapted to a wide variety of habitats growing at 400 m.a.s.l. up to 2700 m.a.s.l. and with different annual rainfall varying from 75 to 1400 mm per year [51]. They show high diversity in morphological traits such as plant height, tiller number and spike length, and physiological traits such as number of days to heading [52].

The early diploid *Aegilops* species have most likely originated in Transcaucasia like the first hexaploid wheat [50,51]. Most *Aegilops* spread to the west except the species carrying the D-genome which spread into Eastern Asia [36,51]. The distribution of wild relatives in the Fertile Crescent partly overlaps with regions where WDV has been detected in Turkey, Syria and Iran [15–17].

The wild relatives of the *Aegilops* genus carry many genes of resistance to fungi and other pathogens and have been used as genetic sources for improvement of rust and powdery mildew resistance in bread wheat ([53] and references therein). In addition, variation in resistance has also been found in *Ae*. *tauschii* and *T*. *monococcum* to *Soil-borne wheat mosaic virus* (SBWMV) [54–56] and in *Ae*. *geniculata* to *Barley yellow dwarf virus* (BYDV) [57]. It has also been shown that the *Aegilops* species *Ae*. *caudata*, *Ae*. *ovata* and *Ae*. *triuncialis* responded with a milder form of symptoms to WDV infection in comparison to spring wheat [18].

The host-pathogen system involving both wild and domesticated wheat provides a unique opportunity to investigate the effect of crop evolution on pathogen resistance. The overlap of the distribution of wild wheat relatives and WDV over a large geographical scale in the Fertile Crescent and adjacent areas suggests that the virus and the wild wheat populations are interacting and co-evolving. The intensity of selection may vary across sites and in time, which have been seen in a wide range of species interactions ([58] and references therein). Among several factors, the virulence of WDV, the population density of the vector, and the physical environment (temperature) may strengthen or weaken the selection locally. We therefore hypothesize that there is a genetic variation in susceptibility to WDV among wild wheat relatives in the Fertile Crescent region. We are also testing the hypothesis that the domestication and human selection, and other genetic bottlenecks during wheat evolution such as natural hybridization and polyploidization events have had a negative impact on the resistance to WDV.

To test our hypotheses we studied the response of one accession each of thirteen wild wheat relatives and five domesticated wheat taxa when exposed to WDV-carrying leafhoppers (Table 1, S1 Table). These accessions are from the Fertile Crescent and adjacent areas (Fig 2). The different taxa have had a direct or indirect role in the evolution of wheat (Fig 1). Interestingly, when the plants were evaluated for various traits associated with WDV resistance, we found three different response patterns. The variation in response was, however, not associated with whether the plants were domesticated or wild. Thus, our findings do not support the assumption that evolutionary processes such as natural hybridization followed by polyploidization and domestication have had an influence on the resistance to WDV in wheat. Instead the variation in susceptibility found in this study may be explained by differences in intensity of natural selection in different geographical areas, shaping the coevolutionary interaction between WDV and the wild wheat relatives in the Fertile Crescent and adjacent areas.

**Table 1:** WDV content in exposed plants within each species the wild and domesticated groups.

**Species**	**Mean**	**95% CI** ^a^
***Aegilops comosa***	0.90	0.35
***Aegilops cylindrical***	1.15	0.28
***Aegilops juvenalis***	1.25	0.23
***Aegilops searsii***	0.83	0.23
***Aegilops sharonensis***	1.06	0.18
***Aegilops speltoides***	0.85	0.33
***Aegilops tauschii***	0.88	0.17
***Aegilops triuncialis***	0.77	0.15
***Aegilops umbellulata***	0.57	0.24
***Amblyopyrum muticum***	1.29	0.38
**Wild einkorn**	1.24	0.38
**Wild emmer**	0.95	0.39
***Triticum urartu***	1.09	0.16
**Bread wheat**	1.06	0.50
**Spelt wheat**	1.83	0.39
**Einkorn wheat**	1.31	0.30
**Emmer wheat**	1.65	0.28
**Durum wheat**	1.13	0.54
**Wild-domesticated status**		
**Domesticated**	1.41	0.14
**Wild**	0.99	0.06
**Controls**		
**Positive (WDV infected source plant)**	1.90	0.33

WDV content is measured as the absorbance at 405 nm using DAS-ELISA.

^a^95% confidence interval.

## Materials and Methods

Thirteen wild and five domesticated wheat taxa were used (Fig 1). They were selected based on their direct or indirect role in the evolution history of wheat [36,45,46] and the co-occurrence with WDV mainly within the Fertile Crescent and adjacent areas. The wild relatives included *Amblyopyrum muticum*, nine *Aegilops* species and three *Triticum* species. These species have different genome types and ploidy levels. The domesticated wheat taxa were the diploid cultivated einkorn, the tetraploids cultivated emmer and durum, and the hexaploids spelta and bread wheat. Seeds of these taxa, except the bread wheat cultivar, were provided by the International Center for Agricultural Research in the Dry Areas (ICARDA), Aleppo, Syria. One accession of each taxon was studied. Their accession numbers and origins are given in S1 Table. The winter wheat cultivar Tarso is derived from crosses between the winter wheat cultivar Taras and the breeding line Hadmerslebener 13313–80 and was released commercially by the breeding company Lantmännen SW Seed AB in 1994.

### *Wheat dwarf virus*

The WDV sources used for inoculation in our study originate both from infected bread wheat plants and the leafhopper vector *P*. *alienus*. These WDV sources were collected in three wheat fields in central Sweden (N 60.0022, E 17.5383; N 59.8384, E 17.7914; N 59.7031, E 17.6994). Sequence analyses of complete genomes have verified that the WDV isolates in this culture are closely related to the WDV isolates previously identified in Sweden and belong to strain WDV-E [59].

### Vector

The leafhopper vectors were collected using sweep nets in June and July 2010. The species identification was done using 10 x magnifier glasses. *P*. *alienus* leafhoppers were reared in nylon mesh covered cages (17 cm x 13 cm x13 cm) on wheat source plants not used in the trial. The plants were grown in ordinary potting soil (Weibulls Horto AB) and the cages were kept in a greenhouse with 16/8 hrs day/night photoperiod and 20/18°C day/night temperature. The first generation of source plants was collected in WDV affected wheat fields and was confirmed by double antibody sandwich enzyme-linked immunosorbent assay (DAS-ELISA, see below) to contain the virus. New healthy wheat plants grown from seeds were regularly replacing old infected ones inside the cages. The new plants were infected by viruliferous leafhoppers and in turn newly hatched and virus-free nymphs acquired WDV from infected wheat plants while feeding on them. This regenerating host-vector-virus system was successfully developed and a population size of several hundred leafhoppers has been maintained since 2009.

### Exposure to viruliferous leafhoppers

Seeds of the different accessions were sown in a mixture of ordinary potting soil (Weibulls Horto AB) and sand of 0.5 mm grain size (Rådasand AB, 1:5 proportions) in a growth chamber with 16/8 hrs day/night photoperiod and 22/20°C day/night temperature. When the plants reached the 2^nd^ leaf stage they were transferred to 2L pots with the same mixture of potting soil and sand and placed in a greenhouse (16/8 hrs day/night photoperiod and 20/18°C day/night temperature). One plant was placed inside a cage (17 cm x 13 cm x 13 cm) covered by nylon fabric with fine mesh avoiding the escape of leafhoppers. When the plants were at the 3^rd^ leaf stage, three nymphs and two adult leafhoppers were transferred to each of the exposed plants by aspirators (insect-collecting tool). After seven days we removed the insects from the cages using the same tool. To make sure that all insects were removed the plants were also treated by the insecticides Pirimor and Confidor (Imidacloprid) at the same time as the cages were dismantled. The number of living insects in each cage was counted in the middle of the inoculation period and if necessary additional insects were transferred to keep the number of insects the same for each exposed plant. Prior to the experiment the nymphs and adult leafhoppers were feeding on infected wheat source plants for a minimum of three days to acquire WDV. These plants were confirmed to carry a high level of WDV using DAS-ELISA.

### Block trial

The plants were grown in a complete randomized block design with 6 blocks. Each block consisted of two plants of each of the 18 accessions. One of the two plants was exposed to viruliferous leafhoppers for seven days as described above, while the other plant of the same accession was not. We randomized the exposed and non-exposed plants of the 18 accessions in each block using the software Research Randomizer (www.randomizer.org). The randomization of accessions and treatments in each of the six blocks enabled us to estimate the block effect and also to reduce the environmental effect on the variation of the studied phenotypes among accessions.

### Evaluated traits

The exposed and non-exposed plants were studied for four traits associated with response to WDV infection. We measured the plant height (base of the stem to the leaf tip of the longest leaf), the total number of tillers, total number of leaves, and percentage of leaf chlorosis (ratio of chlorotic leaves and total number of leaves) 28 days after the end of the exposure to viruliferous leafhopper. A leaf was considered chlorotic if at least 50% of the leaf was yellow. Investigating all leaves on each plant for chlorosis made it possible to assess the whole plant response to WDV infection. The number of tillers and leaves were also measured at a second date, 98 and 112 days after the end of exposure. From now on, we refer to 28 dpi (days after post-inoculation) as the first time point, and 98 dpi and 112 dpi as the second time point for analysis of the different traits. At harvest, 16 weeks after the start of the experiment (112 dpi), each plant was checked for survival (scored as 0 = dead or 1 = alive). A plant was considered dead when most of the leaves where necrotic and/or wilted.

**Fig 3:**
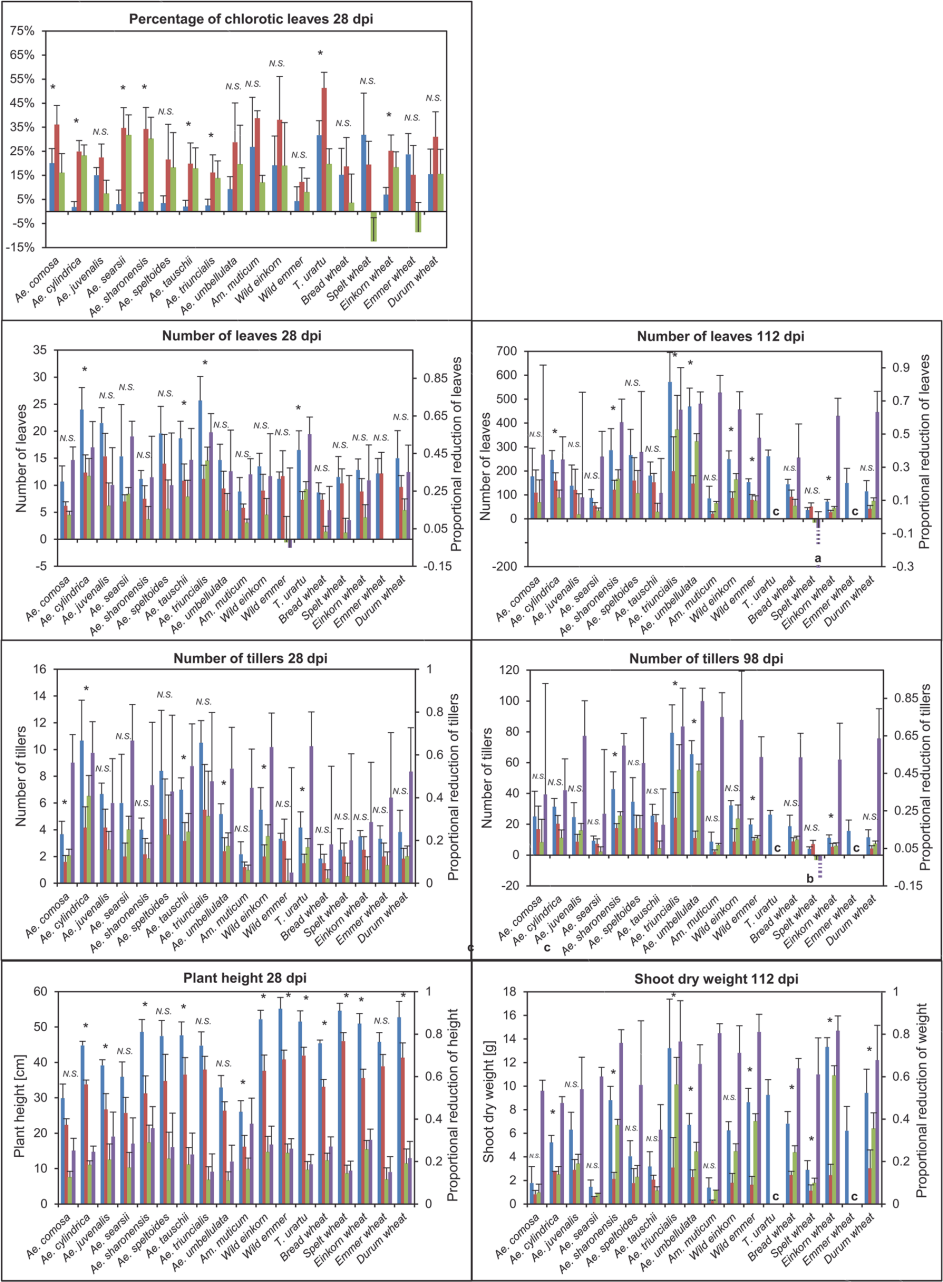
Plant response in wild and domesticated species. BLUE = Mean values and 95% confidence interval (CI) of non-exposed, and RED = exposed plants, GREEN = absolute reduction, PURPLE = proportional reduction of the *Aegilops* and *Triticum* taxa for the studied traits. Pairwise comparison (t-test) between non-exposed and exposed plants with Bonferroni correction. dpi refers to days after the end of exposure to viruliferous leafhoppers. * = p<0.05, N.S. = no significance, ^a^Mean = -39.8%, 95% CI = 44.0, ^b^Mean = -75.0%, 95% CI = 65.7%. ^c^No surviving plants.

As the study comprised accessions from both wild and domesticated taxa with different ploidy level, genome types and degrees of relatedness, variation in the constitutive developmental patterns and morphology was expected. We have therefore chosen to compare the performance of each accession in both exposed and non-exposed control conditions. The response was measured both as the absolute reduction

$$<Display\_Math>{\overset{-}{x}}_{c}-\;{\overset{-}{x}}_{e}\;=\;\frac{1}{n}\sum_{i\;=\;1}^{n}\left({{\overset{-}{x}}_{c}-x}_{i}\right)$$ 1

and as the proportional reduction

$$<Display\_Math>\frac{{\overset{-}{x}}_{c}-\;{\overset{-}{x}}_{e}}{{\overset{-}{x}}_{c}}\;=\;\frac{1}{n}\sum_{i\;=\;1}^{n}\left({{\overset{-}{x}}_{c}-x}_{i}\right)/\;{\overset{-}{x}}_{c}$$ 2

between the two treatments for each trait, where ${\overset{-}{x}}_{c}$ is the average in the non-exposed control condition, ${\overset{-}{x}}_{e}$ the average in the exposed condition and $x_{i}$ the value for the exposed individual *i*. It is very important to distinguish between the two viewpoints since they can give very different results. For example, an accession may have a small absolute reduction in leaf number relative to other accessions, indicating a weak response to WDV. However, the same accession might show a larger proportional reduction for the same trait due to a small number of leaves formed in the control condition as seen for example in einkorn wheat for leaf number at 112 dpi and *Am*. *muticum* for shoot dry weight (Fig 3).

### ELISA

Virus infection of the exposed and non-exposed plants was analyzed by DAS-ELISA. This method has become the standard for detection and quantification of virus content in large number of samples, especially in agricultural crops. We sampled young leaves from all exposed and non-exposed plants at 28 dpi. Virus content was not analyzed at the later growth stage at 98 and 112 dpi due to large differences in plant morphology among wild and domesticated accessions and thereby difficulties in identifying the youngest leaves. Leaves sampled at 28 dpi were kept at -20°C prior to the testing for WDV infection. The plant sap was extracted from 50 mg leaf tissue in 400 μl phosphate buffer (pH 7.4). The samples were diluted 20 times in extraction buffer and analyzed by DAS-ELISA according to Loewe Biochemica GmbH protocol No. 07082 [60]. The plant sap samples were applied onto 96-well microplates of Microlon 600 type and with flat bottoms (Greiner Bio-One) precoated with anti-WDV IgG. The samples were analyzed at 405 nm wavelength in a Benchmark Microplate Reader (Bio-Rad) after 2 hours of incubation with alkaline phosphatase-conjugated anti-WDV IgG and substrate. To verify accuracy and comparison in the tests we included as positive controls 1:20 dilutions of plant sap from a WDV infected bread wheat source plant. Moreover, we used 1:20 dilutions of sap from the non-exposed plants in the trial, and samples only with extraction buffer as negative controls.

### Statistical analysis

Residuals for each trait were checked for deviations from normality. Traits with residuals showing normal distribution were analyzed with a mixed proc model for two-way analysis of variance (ANOVA). Plant height, shoot dry weight, number of tillers at 28 dpi and 98 dpi, number of leaves at 28 dpi and 112 dpi, and percentage of chlorosis showed normally distributed residuals and were therefore analyzed with two-way ANOVA. However, only the absorbance values for WDV content in the exposed plants showed normally distributed residuals and thus analyzed with one-way ANOVA. In addition, difference in mortality, showing binominal distribution, was analyzed by Chi-square test. The plants were grouped in two ways, according to 1) species and 2) whether they are wild or domesticated accessions. These two groups were analyzed separately. We used block as a random factor, and species, wild-domesticated status and treatment (exposed or non-exposed) as fixed factors. We analyzed the following interactions: species, treatment and block, and wild-domesticated status, treatment and block. A t-test was used for pairwise comparisons between the two treatments in each species and correlation among traits was analyzed by simple linear regression. These analyses were done with the statistical software JMP ver. 9 (SAS Institute Inc., Cary, NC, USA).

## Results

### WDV content

In DAS-ELISA tests, the non-exposed plants for all accessions showed low absorbance values (mean = 0.125, 95% CI = 0.002). All plants exposed to viruliferous leafhoppers, except one replicate of *Ae*. *umbellulata*, showed at least twice the absorbance value of the negative control and the non-exposed plants of the corresponding species. These plants were considered positive and infected by WDV. Significant difference in absorbance values for infected plants was found among species (p<0.0001, one-way ANOVA). *Ae*. *umbellulata* showed the lowest mean value (one negative sample excluded), while the domesticated spelt and emmer wheat had the highest WDV content (Table 1).

Based on pairwise comparisons of infected plants between species the domesticated spelt wheat had significantly higher mean absorbance value than seven of the wild species (Tukey’s HSD test, S2 Table). Comparing the wild and domesticated groups a significant difference in mean absorbance value between the groups (p<0.05, t-test) was found, and the mean value was higher in the domesticated group (Table 1).

### Trait variation among non-exposed plants

**Fig 4:**
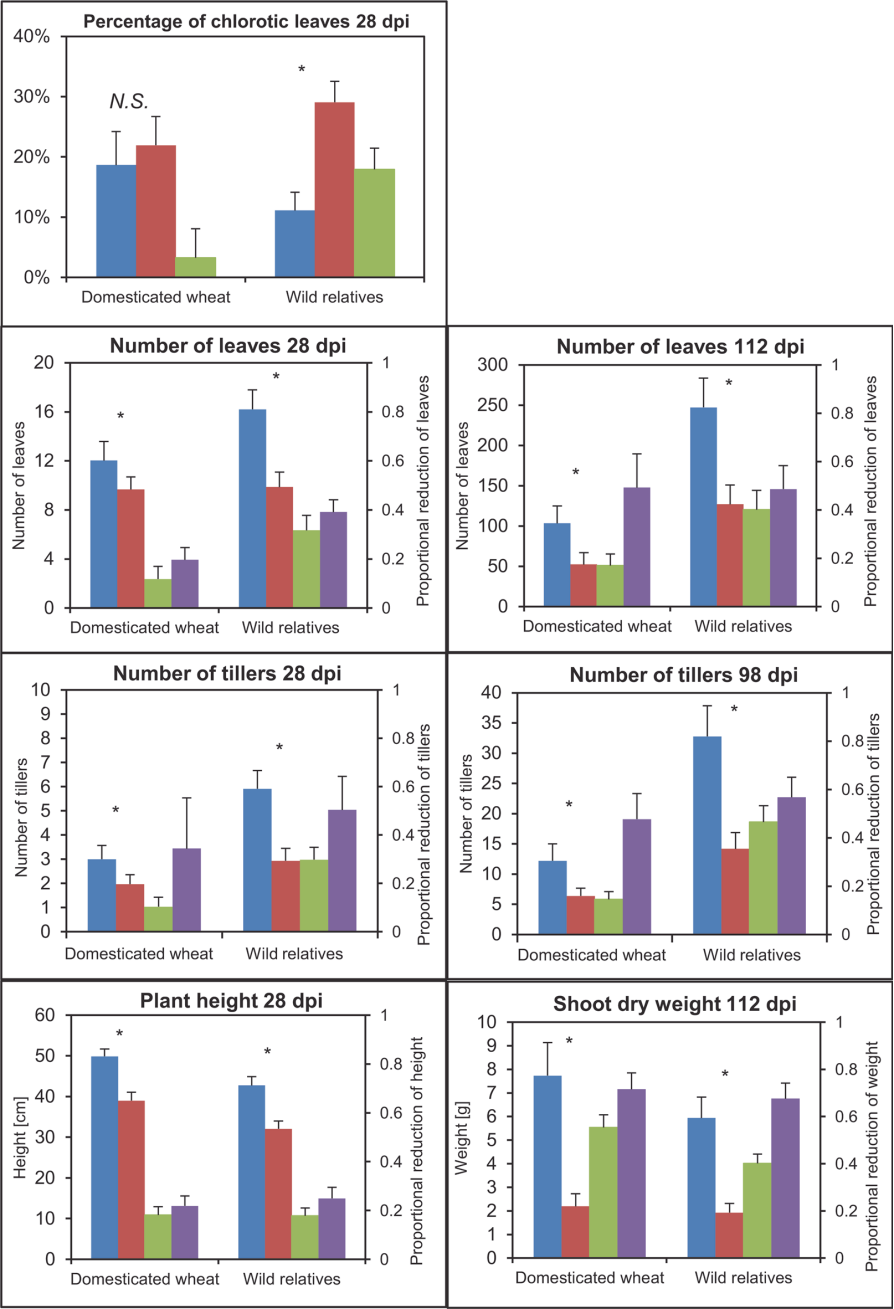
Plant response in the wild and domesticated groups. BLUE = Mean values and 95% confidence interval (CI) of non-exposed, and RED = exposed plants, GREEN = absolute reduction, PURPLE = proportional reduction of the wild and domesticated groups for the studied traits. Pairwise comparison (t-test) between non-exposed and exposed plants with Bonferroni correction. dpi refers to days after the end of exposure to viruliferous leafhoppers. * = p<0.05, N.S. = no significance.

The non-exposed plants showed a large variation among species for all growth traits (Fig 3). The wild species had a significantly lower plant height and shoot dry weight, but a higher number of tillers and leaves for both time points than the domesticated taxa (p<0.05, t-test for all traits, Fig 4). We found also a significant block effect for number of tillers at 28 dpi and number of leaves at 28 dpi (p<0.05, one-way ANOVA), showing a variation of non-exposed plants within accessions. This variation could be a result of environmental variation in the greenhouse and/or the genetic variation within the accessions since the wild accessions, in particular, may not have gone through many generations of selfing. However, no significant block effect was found for exposed plants.

### Effect of treatment

Treatment had a significant effect on all traits (Table 2). The exposed plants showed significantly lower plant height, shoot dry weight and leaf and tiller number at the different time points than the virus-free plants within the domesticated and wild plant groups (Fig 4). The exposed plants within the wild group had also higher percentage of leaf chlorosis, while the exposed and non-exposed domesticated plants were not significantly different. However, pairwise comparisons between exposed and non-exposed plants within each species showed non-significant differences for several species (Fig 3). Most domesticated species showed non-significant differences for percentage of leaf chlorosis and number of leaves and tillers at the two time points, while a larger variation in response was found among the wild species. The mortality was significantly higher in the exposed plants than in the non-exposed plants (Table 3; p<0.05, χ^2^-test,). However, only half of the species was affected. Notable is that all exposed plants of *T*. *urartu* and cultivated emmer died before the harvest at 112 dpi. Also *Ae*. *juvenalis* and wild einkorn showed high mortality. No significant difference was found in mortality between the domesticated and wild groups (p<0.58, χ^2^-test).

**Table 2:** Two-way ANOVA results showing the effects of treatment (exposed or non-exposed), species and their interactions as well as the effect of treatment, wild/domesticated status and their interactions on the studied traits.

**Trait**	**Source**	**F**	**Source**	**F**
**Plant height (28 dpi)**	Treatment	341.3***	Treatment	71.8***
	Species	23.7***	Wild/domesticated	15.9***
	Treatment x species	1.5	Treatment x wild/domesticated	0.0
**Leaf chlorosis (28 dpi)**	Treatment	74.8***	Treatment	22.9***
	Species	4.7***	Wild/domesticated	5.8*
	Treatment x species	2.8***	Treatment x wild/domesticated	10.9**
**Number of leaves (28 dpi)**	Treatment	92.1***	Treatment	29.4***
	Species	9.5***	Wild/domesticated	13.8***
	Treatment x species	2.9***	Treatment x wild/domesticated	6.1*
**Number of tillers (28 dpi)**	Treatment	91.8***	Treatment	28.0***
	Species	11.9***	Wild/domesticated	29.9***
	Treatment x species	2.5**	Treatment x wild/domesticated	6.5*
**Number of leaves (112 dpi)** ^**a**^	Treatment	63.0***	Treatment	13.1***
	Species	20.0***	Wild/domesticated	28.5***
	Treatment x species	4.7***	Treatment x wild/domesticated	3.3
**Number of tillers (98 dpi)** ^**a**^	Treatment	110.2***	Treatment	21.1***
	Species	24.5***	Wild/domesticated	35.2***
	Treatment x species	7.4***	Treatment x wild/domesticated	5.5*
**Shoot dry weight (112 dpi)** ^**a**^	Treatment	236.1***	Treatment	74.6***
	Species	29.8***	Wild/domesticated	10.9**
	Treatment x species	9.9***	Treatment x wild/domesticated	4.2*

^a^Dead plants were not included in the analysis.

* = p<0.05,

** = p<0.01,

*** = p<0.001.

**Table 3:** Plant mortality rate at 112 days after the end of exposure to virouferious leafhoppers.

**Species**	**Treatment**	
	**Exposed**	**Non-exposed**
***Aegilops comosa***	0.00	0.00
***Aegilops cylindrica***	0.00	0.00
***Aegilops juvenalis***	0.67	0.17
***Aegilops searsii***	0.33	0.00
***Aegilops sharonensis***	0.00	0.00
***Aegilops speltoides***	0.00	0.00
***Aegilops tauschii***	0.00	0.00
***Aegilops triuncialis***	0.00	0.00
***Aegilops umbellulata***	0.33	0.00
***Amblyopyrum muticum***	0.50	0.17
**Wild einkorn**	0.67	0.00
***Triticum urartu***	1.00	0.00
**Wild emmer**	0.00	0.00
**Einkorn wheat**	0.00	0.00
**Bread wheat**	0.17	0.00
**Spelt wheat**	0.17	0.00
**Emmer wheat**	1.00	0.00
**Durum wheat**	0.50	0.00

### Variation in response

To determine if there is a variation in susceptibility and resistance to WDV among species as well as the domesticated and wild status groups, we were also focusing on the treatment x species interactions and the treatment x domesticated/wild status. Both the treatment x species and the treatment x domesticated/wild status interactions had significant effect on shoot dry weight, number of tillers at 28 and 98 dpi, number of leaves at 28 dpi, and percentage of chlorotic leaves (Table 2), showing that the species differed in the response to WDV infection. In addition, significant effect was found in the treatment x species interaction for number of leaves at 112 dpi, while no effect was found in the treatment x domesticated/wild status interactions. The variation in response among species is supported by the above pairwise comparisons between treatments. However, all taxa responded in a similar way regarding plant height since no significant interaction was found between treatment x species or between treatment x domesticated/wild status.

#### Leaf chlorosis

Surprisingly, cultivated spelt and emmer wheat showed a lower percentage of chlorotic leaves in the WDV infected plants compared to the non-infected (Fig 3). All other species showed an increase in chlorosis. However, the increase was least severe in *Ae*. *juvenalis* and wild emmer and the bread wheat cultivar Tarso. The highest increase in chlorosis was found in the wild *Ae*. *sharonensis* and *Ae*. *searsii*. The wild plant group showed a significantly larger increase in percentage of chlorotic leaves in the infected plants compared to the domesticated group (p<0.05, t-test; Fig 4).

#### Leaf number at 28 dpi

The leaf number in cultivated emmer and its ancestor wild emmer wheat was not negatively affected by the WDV infection considering both the absolute and proportional reduction (Fig 3). The WDV infection had also a minor effect on cultivated spelt and the bread wheat cultivar Tarso. The highest proportional reduction was found in three wild species *T*. *urartu*, *Ae*. *triuncialis* and *Ae*. *searsii*. In fact, the absolute and proportional reductions in leaf number were most severe in the wild plant group (p<0.05, t-test; Fig 4).

#### Leaf number at 112 dpi

As expected most species were more severely affected at 112 dpi compared to 28 days (Fig 3). In fact, all plants of *T*. *urartu* and cultivated emmer wheat showed a very slow and stunted growth and died before 112 dpi. However, interestingly, the exposed plants of spelt showed an increase in leaf number. WDV infection had also a relatively small effect on *Ae*. *tauschii* and *Ae*. *juvenalis*. *Ae*. *triuncialis* and *Ae*. *searsii*, which were among the most severely affected species at 28 dpi, differed in response at 112 dpi. *Ae*. *searsii* showed a milder response to WDV at 112 dpi than at 28 dpi, while *Ae*. *triuncialis* had a high absolute and proportional reduction also at 112 dpi. The wild plant group showed a higher absolute reduction also at 112 dpi than the domesticated plants (p<0.05, t-test; Fig 4). The proportional reduction was, however, about the same in both groups.

#### Tiller number at 28 dpi

We found the largest proportional reduction in tiller number at 28 dpi in the wild species *Ae*. *searsii*, *Ae*. *cylindrica*, *T*. *urartu*, and the wild einkorn (Fig 3). In addition, *Ae*. *cylindrica* and *Ae*. *triuncalis* showed a large absolute reduction. Wild emmer, the bread wheat cultivar Tarso and spelt wheat had the lowest absolute and proportional reduction.

#### Tiller number at 98 dpi

Interestingly, the exposed plants of spelt showed a notable increase in tiller number (Fig 3). Considering both the absolute and the proportional reduction in tillers the wild species *Ae*. *tauschii* and *Ae*. *searsii* showed the weakest response. Based on the proportional reduction, *Ae*. *umbellulata* was among the most affected species. *Ae*. *umbellulata* showed also a large absolute reduction together with *Ae*. *triuncalis*. The domesticated plant group had significantly lower absolute reduction compared to the wild plants at both time points (p<0.05, t-test; Fig 4). However, the two groups did not differ significantly in the proportional reduction. The difference between the result of the absolute and proportional reductions is most likely due to the large difference in the total number of leaves between the wild and domesticated plants.

#### Shoot dry weight

The proportional reduction in shoot dry weight was less than 50% in *Ae*. *tauschii* and *Ae*. *cylindrica*, while all the other accessions showed a reduction between 53% to 83% (Fig 3). Shoot dry weight of *T*. *urartu* and cultivated emmer was not analyzed since all plants of both species died before 112 dpi. The change in absolute values was noticeably large in the wild *Ae*. *triuncalis* and cultivated einkorn. The absolute reduction was significantly higher in the domesticated plant group (p<0.05, t-test; Fig 4). No significant difference in the proportional reduction in shoot dry weight was shown between the two groups.

#### No correlation between response to WDV and growth habit

The variation in response to WDV measured both as absolute and proportional reduction was independent of the variation among species in the non-exposed condition for all traits studied (R^2^<0.16 for each of the studied traits). This indicates that the response measured as the absolute and proportional reduction is independent of the growth habit of the species under non-exposed condition.

#### Variation in response over time

**Fig 5:**
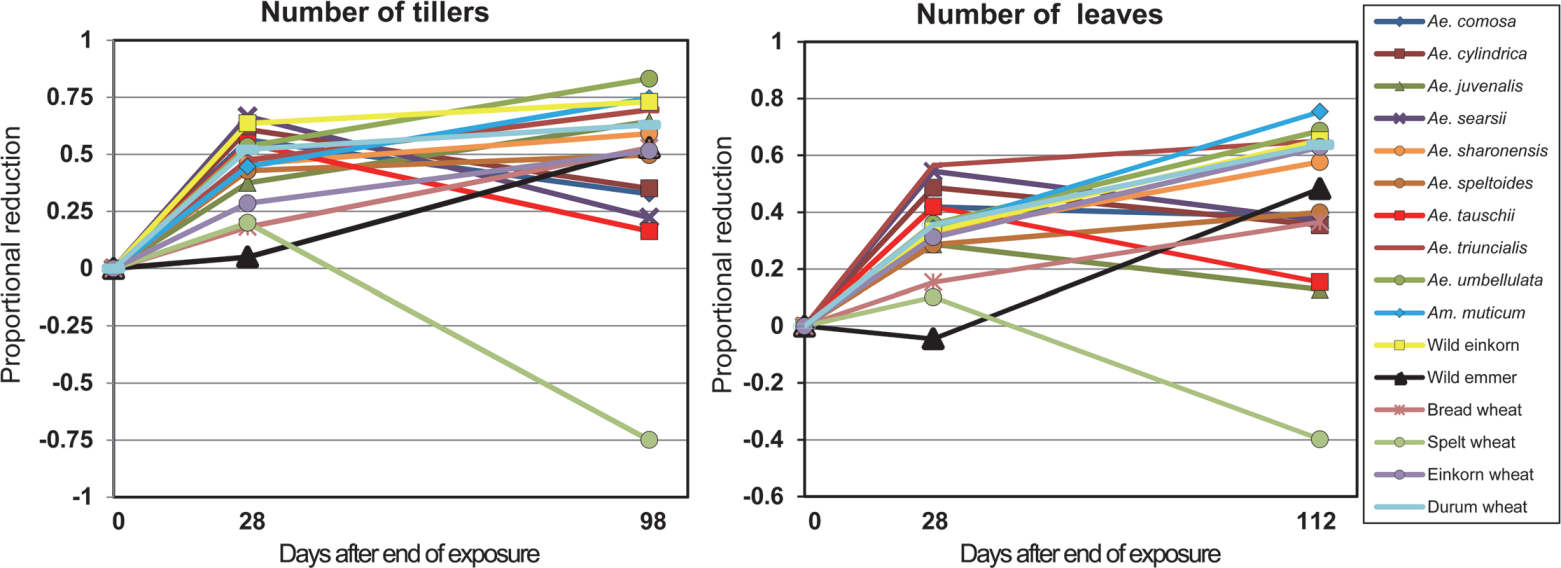
Proportional reduction of leaf and tiller number between non-exposed and exposed plants of wild and domesticated species measured at two time points. All exposed plants of *T*. *urartu* and cultivated emmer were dead at the second time point and not included in the figure.

Comparing the different time points the response pattern in leaf number differed among species (Table 4, Fig 3 and 5). For example, the WDV infection had no effect on cultivated emmer at 28 dpi, whereas all emmer plants were dead at 112 dpi (Table 3). In addition, the exposed plants of wild emmer showed a small increase in leaf number at the first measurement, but an almost 50% reduction 12 weeks later. On the contrary, the *Aegilops* species such as *Ae*. *tauschii* and *Ae*. *searsii* showed a much less proportional reduction in leaf number at the second time point compared to the first. While WDV infection had a small reduced effect on cultivated spelt, at 28 dpi spelt showed a striking increase in leaf production at 112 dpi. Other species such as *Ae*. *triuncialis* and *Ae*. *umbellulata* were highly affected by the WDV infection at both time points.

**Table 4:** Ranking of the studied species based on their response to WDV infection.

	**Chlorosis(28 dpi)**	**Height(28 dpi)**		**Leaves(28 dpi)**		**Tillers(28 dpi)**		**Leaves(112 dpi)**		**Tillers(98 dpi)**		**Weight(112 dpi)**	
**Species**	**A**	**P**	**A**	**P**	**A**	**P**	**A**	**P**	**A**	**P**	**A**	**P**	**A**
***Ae*. *comosa***	9	9	4	13	13	14	9	7	8	4	7	3	2
***Ae*. *cylindrica***	16	8	9	15	15	15	18	4	11	5	10	2	7
***Ae*. *juvenalis***	4	16	13	6	6	5	10	2	2	12	11	4	8
***Ae*. *searsii***	18	14	8	16	16	18	16	6	4	3	2	6	1
***Ae*. *sharonensis***	17	17	18	8	8	9	7	10	14	10	14	12	13
***Ae*. *speltoides***	11	11	14	5	5	7	14	8	12	6	12	5	6
***Ae*. *tauschii***	10	7	10	14	14	13	15	3	3	2	3	1	3
***Ae*. *triuncialis***	7	2	2	18	18	10	17	13	16	13	16	13	15
***Ae*. *umbellulata***	14	5	1	12	12	12	12	15	15	16	15	9	9
***Am*. *muticum***	6	18	7	10	10	8	4	16	7	15	5	14	4
**Wild einkorn**	13	13	16	9	9	16	13	14	13	14	13	11	11
**Wild emmer**	5	10	15	1	1	1	1	9	10	9	9	15	14
***Triticum urartu***	15	4	6	17	17	17	11	-^a^	-^a^	-^a^	-^a^	-^a^	-^a^
**Bread wheat**	3	12	12	4	4	2	2	5	6	8	8	8	9
**Spelt wheat**	1	3	5	3	3	3	3	1	1	1	1	7	5
**Einkorn wheat**	12	15	17	7	7	4	5	11	5	7	4	16	16
**Emmer wheat**	2	1	3	2	2	6	6	-^a^	-^a^	-^a^	-^a^	-^a^	-^a^
**Durum wheat**	8	6	11	11	11	11	8	12	9	11	6	10	12

Number 1 signifies the species with the lowest proportional (P) or absolute (A) reduction and number 18 the species with the largest reduction for each trait. dpi refers to the end of the exposure time to viruliferous leafhoppers.

As for the leaf number, we also observed a difference in response pattern in tiller number between the two time points. Most notable is the much weaker response at 98 dpi compared to 28 dpi for *Ae*. *tauschii* and *Ae*. *searsii*. Even though the bread wheat cultivar Tarso and wild emmer wheat were among the species with lowest proportional and absolute reduction in tiller number at the first measurement, they were much more severely affected at 98 dpi. *Ae*. *umbellulata* and wild einkorn showed a strong response to WDV at both time points.

Taking also chlorosis, plant height, shoot dry weight and mortality in consideration, cultivated spelt was among the least affected species for all studied traits and time points (Table 4). In contrary, the wild species *T*. *urartu*, *Ae*. *triuncialis*, *Ae*. *umbellulata* and wild einkorn showed a strong response. Interestingly, some of the wild species such as *Ae*. *tauschii*, *Ae*. *searsii* and *Ae*. *comosa* were severely affected in most traits measured at the first time point, while they were among the least affected species at the later measurements. The bread wheat cultivar Tarso, showed a different level of response for various traits and time points.

#### Correlation between traits, and between traits and ELISA-values

Based on simple linear regression we found strong positive correlation between number of tillers at 98 dpi and leaves at 112 dpi considering both exposed and non-exposed plants together (S3 Table). A strong positive correlation was also found when the exposed and non-exposed plants were analyzed separately. The same traits also showed a positive correlation at 28 dpi, although weaker, for all the three plant groups analyzed (all plants, exposed plants, and non-exposed plants). However, no strong correlation was found between number of leaves and the other growth traits (height and weight), or between the number of tillers and the two growth traits. Interestingly, there was no evident correlation between any growth trait and ELISA-value. Moreover, chlorosis did not correlate strongly with ELISA-value or any of the growth traits.

## Discussion

Crops are constantly confronted with a wide variety of potential pathogens within their environment. Cultivation of monocultures with genetically uniform plants in dense stands allowing closer contact between plants and vectors has led to efficient transmission of pathogens and evolution of more aggressive strains [61,62]. These strains cause substantial damage to crops but our knowledge to prevent the pathogen infestations is often limited to the use of pesticides, leading to environmental and ecological risks. Aiming for a sustainable agriculture, breeding for disease resistant cultivars is an important component in the process. As strong selection during crop domestication often resulted in loss of genetic diversity and traits not directly selected for such as resistance to pest and diseases, the wild relatives may be the most useful or only genetic sources for introgression of resistance into crops.

We have compared the variation in response to WDV infections in wild and domesticated wheat taxa to test whether the interaction between wild wheat relatives and the virus has coevolved under divergent intensity of selection and led to varying degrees of susceptibility in the host plants. We have also investigated if the genetic bottlenecks created by natural hybridization and polyploidization followed by the domestication process have resulted in a loss of resistance to WDV in wheat.

Aiming to increase the understanding of the variation in response to WDV in an evolutionary history context, one accession of each of thirteen wild and five domesticated species directly or indirectly involved in wheat evolution were selected rather than a larger number of accessions within a few species. The large genetic diversity among the studied species, with different genome type and ploidy level, and from different geographic locations, has most likely increased the chances of variation in intensity of selection and strength of coevolution between the virus and the host plants. The large diversity will therefore increase the ability to identify genetic resources, which are critical for effective breeding programs. Because the within-species variation has not been studied here our results may not be broadly applied to the species level.

We have chosen a number of different traits for the experimental study of symptomatic response such as leaf chlorosis, plant height, shoot dry weight and leaf and tiller number. To our knowledge this is the first study which has investigated the response to WDV infection in wild and domesticated wheat by combining the analyses of leaf chlorosis, different growth traits and WDV content. Chlorosis and reduced growth are commonly associated with WDV infection in wheat and were therefore selected for the investigation of variation in response in wild and domesticated wheat. These symptoms are caused by large changes in cellular and developmental processes in the host plants, but the genetic and cellular mechanisms behind these changes are not well known. However, studies of geminiviruses have demonstrated that they interact with different plant proteins leading to a transition from normal host growth processes to altered metabolic pathways and defense responses ([63,64] and references therein). These proteins are joined in different protein complexes. Some of these complexes are players in cellular processes known as RNA silencing. This antiviral response restricts the accumulation and movement of viruses within the plant. However, as a way to escape the RNA silencing defense the viruses have evolved RNA silencing suppressor proteins (RSS). Several RSSs have been described for geminiviruses [65], and recently two replication-associated proteins encoded by WDV were shown to suppress the RNA silencing system [66,67]. The RSSs are not only involved in antiviral defense, but are also affecting the growth and development of the plant by interfering with cellular processes regulated by RNA silencing [63,68,69]. In another virus-plant interaction it was recently shown that the coat protein of the *Cucumber mosaic virus* (CMV; family *Bromoviridae*) and the encoded RNA silencing suppressor 2b protein repress the expression of chloroplast and photosynthesis related genes [70]. This reduces the number of chloroplast thylakoid membranes and causes chlorosis in CMV infected tobacco leaves. A decrease of photosynthesis may in turn initiate respiration and other processes involved in the host plant defense [71]. Callose deposition of saccharide callose at the plasmodesmata channels in the cell walls is another defense response that restricts the cell-to-cell movement of viruses within the plant [72]. At the same time the callose deposition may also delay photoassimilate export from the infected leaves and restrict phloem transportation followed by chlorophyll breakdown [73].

### Large variation in symptomatic response among accessions

We found a large variation in response to WDV infection in the wild relatives of wheat in leaf chlorosis as well as the different growth traits. The response ranged from increased growth, to modest decrease of growth and no surviving plants. By combining the results from the various traits and time points with the analysis of WDV content we were able to obtain a more complete picture of the variation in response in the different wild and domesticated wheat taxa. Interestingly, the response also changed over time for some accessions, where the plants showed severe symptoms at an early developmental stage but milder symptoms at a later stage and continued to produce new leaves and tillers. We found three different plant response patterns over time (Fig 5). Several of the accessions of the wild species such as *Ae*. *umbellulata*, *Ae*. *triuncialis*, *Am*. *muticum*, wild einkorn and durum wheat showed a continuous reduction in the number of tillers and leaves between the time points. The growth was also severely affected in *T*. *urartu* and no plants were alive at the second time point. Other accessions such as bread wheat, wild emmer and cultivated emmer wheat showed a weak response to the WDV infection at the first time point but a much stronger response at the end of the experiment. In fact, all plants of cultivated emmer wheat died before the end of the experiment. An opposite response was found in the accessions *Ae*. *tauschii*, *Ae*. *cylindrica* and *Ae*. *searsii*, where the plants were severely affected at the first measurement, but showed a relative increase in production of tillers and leaves at the later time point. This increase in production over time was even more pronounced in spelt wheat, where the number of leaves and tillers in exposed plants was higher in comparison with the production in the non-exposed plants. Increased growth of tillers has also been observed in WDV infected winter wheat [19], and in BYDV infected oat and barley plants grown in field trials [74,75]. It has been suggested that increased vegetative production in BYDV infected oat is the result from reduced grain sink strength [76], and reallocation of host resources [77,78]. Spike formation was noted in our study and a reduction in spike formation was found in almost all accessions infected with WDV (S4 Table). However, the reduction was not more prominent in the accessions with no or less affected growth of leaves and shoots such as spelt wheat and *Ae*. *tauschii*. The data of the spike formation have to, however, be treated with caution since some of the studied accessions, including winter bread wheat, may be vernalization-sensitive, producing fewer spikes when the seedlings of these accessions have not been exposed to lower temperatures.

The remission of symptoms found in several *Aegilops* species and spelt wheat has also been observed in dicots infected with geminiviruses such as tobacco, cassava, *Arabidopsis*, pepper, zucchini and melon [79–84]. These decreases of symptoms are associated with a reduction of virus content triggered by the RNA silencing system. We observed medium to high levels of virus content in all studied accessions at 28 dpi. Due to large differences in plant morphology among wild and domesticated accessions and thereby difficulties in identifying the youngest leaves the WDV content was not investigated at the later time point. Sampling of leaves of different ages would most likely have caused a bias in the analysis. We could therefore not investigate if the decrease of symptoms in some *Aegilops* species was associated with a reduction in virus content in the plants. Interestingly, in a preliminary study of a collection of *Ae*. *tauschii* accessions kept at John Innes Centre, Norwich, UK, we observed a reduction of both symptoms and WDV content in some accessions between two different developmental stages. However, in the present study at 28 dpi the severity of symptoms in wild and domesticated wheat was not correlated with the WDV content. Plants with high virus content did not necessarily show strong reduction in growth or high levels of chlorosis. In fact, spelt wheat had the highest WDV content but showed the mildest symptoms among accessions, which may indicate some degree of tolerance against WDV. In addition, the remission of symptoms over time in *Ae*. *tauschii* and some other *Aegilops* species may likewise indicate tolerance. On the other hand, *Ae*. *umbellulata* and *Ae*. *triuncialis* showed very strong response in almost all traits even though they had the lowest WDV content among the studied accessions.

The lack of correlation between WDV content and severity of symptoms in the different *Aegilops* and *Triticum* species may at first be surprising as one may expect that the rate and extent of the virus amplification and movement within the plant will influence the severity of symptoms. It has, however, been shown that even though the RSSs of plant-infecting viruses are involved in the induction of symptoms, symptom severity and virus accumulation are not necessarily correlated [85,86]. The activity of the RSSs may have a stronger effect on the symptomatic responses than the virus content in the plant and may be one of the explanations to the lack of strong correlation between the severity of symptoms and WDV content in the *Aegilop*s and *Triticum* plants.

### No impact of domestication on WDV resistance

To test the hypothesis that domestication and human selection have had a negative impact on the resistance to WDV we compared the response between the two groups, domesticated wheats and their wild relatives. We found no strong evidence that the domesticated wheats suffered from more severe symptoms than the wild relatives. In fact, no significant difference in proportional reduction was found between wild and domesticated wheats for chlorosis and all growth traits except leaf number at dpi 28. These results suggest that the loss of genetic diversity expected by the domestication process and other bottlenecks such as natural hybridization and polyploidization events during wheat evolution have not resulted in a general increase in susceptibility to WDV infection. The reduced genetic diversity caused by these bottlenecks may have been compensated by the hybridization of the different ancestral genomes followed by duplication, resulting in new genetic diversity. In addition, the polyploid nature of tetraploid and hexaploid wheats enables buffering capacity and greater robustness against gene mutations. This allows for a rapid formation of new genetic variation and novel traits [35]. However, the WDV content was significantly higher in the domesticated wheat group. This suggests that the virus amplification and/or movement within the plants in the domesticated group are higher than in the wild plant group. Virus encoded movement proteins (MPs) interact with host proteins to promote virus movement within the plant. Different host proteins have been identified at different subcellular locations and in different virus host-plant interactions ([63] and references therein). In our study all plants have been infected with the same WDV strain. However, not much is known about host proteins interacting with plant virus MPs in grasses. The domesticated and wild wheat species studied differs in genome type and ploidy level. If this diverse plant material would harbor a variation in host proteins, one may ask if specific interactions between WDV and plant proteins could explain the difference in WDV content among the wild and domesticated species.

## Conclusions

Our findings do not support the assumption that evolutionary processes such as natural hybridization followed by polyploidization and domestication have had a strong influence on the resistance to WDV in wheat. Instead other processes such as natural selection may have shaped the coevolutionary interaction between WDV and the wild wheat relatives in the Fertile Crescent and adjacent areas. The arms race involving differences in the intensity of selection imposed on the interaction between the virus and the different host plants has resulted in a variation in response to WDV. Variation in virulence and host susceptibility among populations, geographical regions and related species will create what Thompson [87] has called geographic mosaics of coevolutionary hotspots with strong reciprocal selection and cold spots with relaxed selection. These selection mosaics have been seen in a wide range of species interactions [87]. The variation in susceptibility found among *Aegilops* and *Triticum* species is likely caused by an evolutionary response to variation in selection pressure on the interacting organisms—host plants, virus and insect vector—over time. The influence of the leafhopper vector on the evolution of this interaction is not known. However, the population density of the vector and its efficiency in transferring the virus to the different hosts may vary across sites and thereby further affect the intensity of selection.

Moreover, we found that the accessions of the A genome donor, *T*. *urartu*, the putative B genome donor *Ae*. *speltoides* and the D genome donor of bread wheat, *Ae*. *tauschii*, showed different patterns of response to WDV. Both infected *T*. *urartu* and *Ae*. *speltoides* plants had a continuous reduction in growth, whereas *Ae*. *tauschii* displayed a remission of symptoms over time. Even though our results may not be broadly applied to a species level since only one accession of each ancestral species have been studied, our findings indicate that the susceptibility to WDV may be associated with the genome type and that *Ae*. *tauschii* may be useful as a genetic resource for the improvement of resistance to WDV in bread wheat. From the viewpoint of our results, we suggest further evaluation of different accessions of *Ae*. *tauschii*. In addition, it would be of interest to further investigate the potential of *Aegilops* species, *Ae*. *cylindrica*, *Ae*. *comosa* and *Ae*. *searsii*, as well as spelt wheat as genetic resources for the improvement of WDV resistance in wheat. For a more complete picture of the response to WDV, we propose that different traits associated to WDV infection will be investigated at different plant development stages.

## Supporting Information

S1 Table Studied accessions and their passport data. The passport data were obtained from the SINGER data base, 2009. ^a^N/A = data not available
(DOCX)

S2 Table Tukey’s HDS test (pairwise comparisons) of WDV content in exposed plants in the studied species. Similar letters indicate no significant difference.
(DOCX)

S3 Table Correlation between traits shown as coefficient of determination (R^2^). N-E = non-exposed plants, E = exposed plants, All = All samples (exposed and non-exposed plants).
(DOCX)

S4 Table Average number of spikes in exposed and non-exposed plants of wild and domesticated species. (DOCX)
