# Supplementary material for: Variation in Susceptibility to Wheat dwarf virus among Wild and Domesticated Wheat
Source: PLoS One. 2015 Apr 2;10(4):e0121580. doi: 10.1371/journal.pone.0121580 (PMC4383415; doi:10.1371/journal.pone.0121580)
Supplement: S1 Table — The passport data were obtained from the SINGER data base, 2009. aN/A = data not available (DOCX) [file pone.0121580.s001.docx]

**S1 Table. Studied accessions and their passport data.**

| **Species** | **Accession** | **Coordinates** | **Country** | **Altitude**  **(m.a.s.l.)** |
| --- | --- | --- | --- | --- |
| ***Aegilops comosa*** | IG48600 | N 29.50, E 40.82 | Turkey | 175 |
| ***Aegilops cylindrica*** | IG46976 | N 37.28, E 44.57 | Turkey | 1937 |
| ***Aegilops juvenalis*** | IG48478 | N 37.06, E 42.06 | Syria | 525 |
| ***Aegilops searsii*** | IG47335 | N 32.17, E 35.87 | Jordan | 802 |
| ***Aegilops sharonensis*** | IG47098 | N 38.60, E 27.01 | Turkey | 149 |
| ***Aegilops speltoides*** | IG110778 | N 37.04, E 40.02 | Syria | 525 |
| ***Aegilops tauschii*** | IG46897 | N 36.58, E 45.42 | Iran | 2034 |
| ***Aegilops triuncialis*** | IG116094 | N 36.84, E 36.84 | Turkey | 760 |
| ***Aegilops umbellulata*** | IG47962 | N 38.47, E 39.30 | Turkey | 1581 |
| ***Amblyopyrum muticum*** | IG131314 | N 38.98, E 45.05 | Iran | 1146 |
| ***Triticum aestivum* ssp. *aestivum* cv. Tarso (bread wheat)** |  |  | Germany |  |
| ***Triticum aestivum* ssp. *spelta* (spelt)** | IG127036 | N/A^a^ | USA | N/A^a^ |
| ***Triticum monococcum* ssp. *boeticum* (wild einkorn)** | IG116144 | N 37.27, E 37.52 | Turkey | 720 |
| ***Triticum monococcum* ssp. *monococcum* (einkorn)** | IG45110 | N 38.60, E 27.07 | Turkey | 149 |
| ***Triticum turgidum* ssp. *dicoccoides* (wild emmer)** | IG45725 | N 32.53, E 35.78 | Jordan | 391 |
| ***Triticum turgidum* ssp. *dicoccon* (emmer)** | IG45033 | N 37.57, E 44.2 | Turkey | 2049 |
| ***Triticum turgidum* ssp. *durum* (durum)** | IG82696 | N 37.58, E 38.95 | Turkey | 609 |
| ***Triticum urartu*** | IG110784 | N 37.20, E 42.18 | Syria | 417 |
