# Supplementary material for: Variation in Susceptibility to Wheat dwarf virus among Wild and Domesticated Wheat
Source: PLoS One. 2015 Apr 2;10(4):e0121580. doi: 10.1371/journal.pone.0121580 (PMC4383415; doi:10.1371/journal.pone.0121580)
Supplement: S2 Table — Similar letters indicate no significant difference. (DOCX) [file pone.0121580.s002.docx]

| **Species** |  |  |  | **Mean** |
| --- | --- | --- | --- | --- |
| **Spelt wheat** | A |  |  | 1.83 |
| **Emmer wheat** | A | B |  | 1.65 |
| **Einkorn wheat** | A | B | C | 1.31 |
| ***Amblyopyrum muticum*** | A | B | C | 1.29 |
| ***Aegilops juvenalis*** | A | B | C | 1.25 |
| **Wild einkorn** | A | B | C | 1.24 |
| ***Aegilops cylindrica*** | A | B | C | 1.15 |
| **Durum wheat** | A | B | C | 1.13 |
| ***Triticum urartu*** | A | B | C | 1.09 |
| ***Aegilops sharonensis*** | A | B | C | 1.06 |
| **Bread wheat** | A | B | C | 1.06 |
| **Wild emmer** |  | B | C | 0.95 |
| ***Aegilops comosa*** |  | B | C | 0.90 |
| ***Aegilops tauschii*** |  | B | C | 0.88 |
| ***Aegilops speltoides*** |  | B | C | 0.85 |
| ***Aegilops searsii*** |  |  | C | 0.83 |
| ***Aegilops triuncialis*** |  |  | C | 0.77 |
| ***Aegilops umbellulata*** |  |  | C | 0.57 |

**S2 Table.** **Tukey’s HDS test (pairwise comparisons) of WDV content in exposed plants in the studied species.**
