# Supplementary material for: Variation in Susceptibility to Wheat dwarf virus among Wild and Domesticated Wheat
Source: PLoS One. 2015 Apr 2;10(4):e0121580. doi: 10.1371/journal.pone.0121580 (PMC4383415; doi:10.1371/journal.pone.0121580)
Supplement: S3 Table — N-E = non-exposed plants, E = exposed plants, All = All samples (exposed and non-exposed plants). (DOCX) [file pone.0121580.s003.docx]

|  | **R²** | **Leaves (112 dpi)** | **Tillers**  **(28 dpi)** | **Tillers**  **(98 dpi)** | **Chlorosis (28 dpi)** | **Height**  **(28 dpi)** | **ELISA**  **(28 dpi)** | **Weight (112 dpi)** |
| --- | --- | --- | --- | --- | --- | --- | --- | --- |
| **Leaves (28 dpi)** | **All** | 0.21 | 0.71 | 0.18 | 0.25 | 0.15 | 0.11 | 0.13 |
|  | **N-E** | 0.14 | 0.68 | 0.11 | 0.10 | 0.01 | 0.01 | 0.03 |
|  | **E** | 0.16 | 0.58 | 0.06 | 0.29 | 0.17 | 0.01 | 0.06 |
| **Leaves (112 dpi)** | **All** |  | 0.33 | 0.92 | 0.13 | 0.03 | 0.12 | 0.32 |
|  | **N-E** |  | 0.22 | 0.92 | 0.07 | 0.01 | 0.01 | 0.21 |
|  | **E** |  | 0.51 | 0.87 | 0.07 | 0.03 | 0.07 | 0.46 |
| **Tillers (28 dpi)** | **All** |  |  | 0.29 | 0.27 | 0.01 | 0.13 | 0.11 |
|  | **N-E** |  |  | 0.19 | 0.18 | 0.01 | 0.01 | 0.01 |
|  | **E** |  |  | 0.37 | 0.20 | 0.07 | 0.01 | 0.30 |
| **Tillers (98 dpi)** | **All** |  |  |  | 0.15 | 0.03 | 0.14 | 0.29 |
|  | **N-E** |  |  |  | 0.08 | 0.01 | 0.01 | 0.18 |
|  | **E** |  |  |  | 0.07 | 0.03 | 0.06 | 0.40 |
| **Chlorosis (28 dpi)** | **All** |  |  |  |  | 0.15 | 0.11 | 0.12 |
|  | **N-E** |  |  |  |  | 0.01 | 0.01 | 0.03 |
|  | **E** |  |  |  |  | 0.12 | 0.01 | 0.03 |
| **Height (28 dpi)** | **All** |  |  |  |  |  | 0.13 | 0.33 |
|  | **N-E** |  |  |  |  |  | 0.01 | 0.20 |
|  | **E** |  |  |  |  |  | 0.05 | 0.08 |
| **ELISA (28 dpi)** | **All** |  |  |  |  |  |  | 0.24 |
|  | **N-E** |  |  |  |  |  |  | 0.01 |
|  | **E** |  |  |  |  |  |  | 0.01 |

**S3 Table.** **Correlation between traits shown as coefficient of determination (R²)**.
