# Supplementary material for: Variation in Susceptibility to Wheat dwarf virus among Wild and Domesticated Wheat
Source: PLoS One. 2015 Apr 2;10(4):e0121580. doi: 10.1371/journal.pone.0121580 (PMC4383415; doi:10.1371/journal.pone.0121580)
Supplement: S4 Table — (DOCX) [file pone.0121580.s004.docx]

**S4 Table.** **Average number of spikes in exposed and non-exposed plants of wild and domesticated species.**

|  | |  |  | |  | |  |  |  |  |  |
| --- | --- | --- | --- | --- | --- | --- | --- | --- | --- | --- | --- |
| **Species** | | | **Treatment** | | | |  |  |  |  |  |
|  | | | **Exposed** | | **Non-exposed** | |  |  |  |  |  |
| ***Aegilops comosa*** | | | 0 | | 0 | |  |  |  |  |  |
| ***Aegilops cylindrica*** | | | 0 | | 0 | |  |  |  |  |  |
| ***Aegilops juvenalis*** | | | 45 | | 126 | |  |  |  |  |  |
| ***Aegilops searsii*** | | | 2 | | 21 | |  |  |  |  |  |
| ***Aegilops sharonensis*** | | | 1 | | 8 | |  |  |  |  |  |
| ***Aegilops speltoides*** | | | 3 | | 10 | |  |  |  |  |  |
| ***Aegilops tauschii*** | | | 0 | | 5 | |  |  |  |  |  |
| ***Aegilops triuncialis*** | | | 0 | | 108 | |  |  |  |  |  |
| ***Aegilops umbellulata*** | | | 0 | | 16 | |  |  |  |  |  |
| ***Amblyopyrum muticum*** | | | 0 | | 0 | |  |  |  |  |  |
| **Wild einkorn** | | | 0 | | 0 | |  |  |  |  |  |
| ***Triticum urartu*** | | | 0 | | 1 | |  |  |  |  |  |
| **Wild emmer** | | | 15 | | 21 | |  |  |  |  |  |
| **Einkorn wheat** | | | 31 | | 79 | |  |  |  |  |  |
| **Bread wheat** | | | 0 | | 4 | |  |  |  |  |  |
| **Spelt wheat** | | | 1 | | 2 | |  |  |  |  |  |
| **Emmer wheat** | | | 0 | | 18 | |  |  |  |  |  |
| **Durum wheat** | | | 9 | | 6 | |  |  |  |  |  |
